# AI literacy and vocational students’ complex problem-solving skills: a moderated mediation model of systematic thinking, cognitive load, and perceived school ICT support

**DOI:** 10.3389/fpsyg.2026.1971672

**Published:** 2026-10-28

**Authors:** Maoyong Huang, Meiying Huang, Ting He, Xing Yi, Wenjie Deng

**Affiliations:** Guangdong Polytechnic Normal University, Guangzhou, China

**Keywords:** artificial intelligence literacy, cognitive load, complex problem-solving skills, perceived school ICT support, systematic thinking skills

## Abstract

As technological advancements such as Artificial Intelligence (AI) reshape the landscape of work, career development in vocational education is increasingly oriented toward dynamic, long-term personal growth. In this context, Complex Problem-Solving Skills (CPS) have become a critical competence, reflecting students’ capacity to address ill-structured tasks and adapt to rapidly changing environments. Grounded in Social Cognitive Theory, this study examines the relationship between AI Literacy and students’ self-reported Complex Problem-Solving Skills (CPS) in higher vocational colleges and the cognitive pathways that may help explain this relationship. This study posits that AI Literacy, as a personal agentic factor, is positively associated with self-reported CPS both directly and indirectly through Systematic Thinking Skills and cognitive load, and that perceived school information and communication technology (ICT) support may condition the strength of these associations. To provide temporal separation among the focal measures, a multi-wave survey was administered to 784 students from six higher vocational institutions at three time points. Data were analyzed using SPSS and Mplus. Results indicated that AI Literacy was significantly associated with self-reported CPS, with significant indirect effects estimated through Systematic Thinking Skills and the six-item cognitive-load composite. Notably, the associations between AI Literacy and these cognitive factors were stronger at higher levels of perceived school ICT support. These findings highlight the relevance of AI Literacy and students’ perceptions of ICT support to self-reported CPS and their preparation for AI-integrated workplaces.

## 1 Introduction

Complex problem solving (CPS) is recognized as a core higher-order skill that enables individuals to address unforeseen societal and occupational challenges in the 21st century (Mainert et al., 2019). Traditionally, CPS refers to the ability to identify critical issues, understand dynamic interrelationships, formulate strategies, and adjust action plans in environments characterized by incomplete information and interacting variables (Dörner and Funke, 2017). These capabilities are particularly relevant in vocational education, where students are expected to apply knowledge to changing, practice-oriented, and often ill-structured problems. Such capabilities are also increasingly relevant to students’ preparation for technology-rich learning and future work (Chee et al., 2025). In the present study, CPS is operationalized through students’ self-reports of their perceived problem-solving capabilities rather than through performance on an objective complex problem-solving task.

However, the proliferation of artificial intelligence (AI) is reshaping the practical context in which complex problem solving takes place (Ozer et al., 2025). Emerging forms of human–AI collaboration, particularly those involving generative AI, provide new resources for problem solving while also introducing new cognitive demands. Effective use of AI involves more than asking a system to generate information; users must also evaluate the reliability and limitations of AI outputs and integrate externally generated information into their own judgment and decision-making processes (Marques et al., 2024). Consequently, effective CPS increasingly involves not only independent analytical ability but also the capacity to interact critically and productively with intelligent systems. This shift makes students’ ability to understand, use, and evaluate AI particularly relevant to contemporary problem solving.

Against this background, AI Literacy becomes an important personal capability for students who engage in AI-supported problem solving. From a Social Cognitive Theory perspective, individuals’ ability to approach challenging tasks is partly related to the knowledge, skills, and beliefs they bring to those tasks (Bandura, 1986). AI Literacy captures such capabilities in relation to AI: it encompasses the knowledge and skills required to identify, apply, and evaluate AI technologies, together with the beliefs and judgments involved in their responsible use (Long and Magerko, 2020; Wang et al., 2023). Students with stronger AI Literacy may therefore be better positioned to use AI resources purposefully, evaluate generated information critically, and integrate AI outputs into their own reasoning rather than relying on them uncritically. Consistent with this broader educational relevance, recent research has linked AI-related literacy and use with students’ learning outcomes and academic performance (Shahzad et al., 2024; Singh et al., 2025).

The educational relevance of AI Literacy has therefore received increasing attention, both in discussions of technology-supported learning and in calls to prepare students for AI-rich educational environments (Lu and Xie, 2024; Walter, 2024). More directly, Promma et al. (2025) reported relationships among AI Literacy, Systematic Thinking Skills, and Complex Problem-Solving Skills, indicating that the basic association between AI Literacy and CPS is not entirely unexplored. Related higher-education research has likewise emphasized that transferable meta-skills are relevant to graduate readiness and that their development depends partly on how higher education organizes learning and academic support (Che et al., 2026a). Taken together, these studies establish a useful foundation, but they leave several questions open. In particular, less is known about how AI Literacy is associated with self-reported CPS among vocational college students when multiple cognitive pathways and perceived environmental support are considered together. Cognitive load has received limited attention as an additional cognitive pathway, and perceived school ICT support has rarely been examined as a contextual condition that may alter the strength of these relationships. These questions are especially relevant in higher vocational education, where students are expected to apply technological knowledge in practice-oriented and industry-related problem-solving contexts.

To clarify how AI Literacy may be associated with CPS, the present study focuses on two cognitive factors: Systematic Thinking Skills and cognitive load. First, effective engagement with AI often requires users to define and structure a problem, examine relationships between inputs and outputs, identify and prioritize relevant information, and integrate information from multiple sources before reaching a judgment. These activities correspond closely to the dimensions of Systematic Thinking Skills examined in this study. Related systems-thinking research similarly emphasizes attention to interrelationships among elements within a broader problem structure (Assaraf and Orion, 2005). Thus, higher AI Literacy may support a more structured and systematic approach to complex problems. Second, AI Literacy may also be relevant to the amount of cognitive effort students experience when using AI-supported resources. Because working-memory resources are limited (Sweller, 2024), students who are unfamiliar with AI tools may devote substantial effort to basic operations, such as learning software functions or repeatedly adjusting prompts, leaving fewer cognitive resources available for reasoning about the core problem. In contrast, greater familiarity with AI may allow routine operations such as information retrieval and classification to be handled more efficiently. These two cognitive processes therefore provide complementary explanations for how AI Literacy may be related to self-reported CPS.

The potential value of these cognitive processes may also depend on the environment in which students use AI. Social Cognitive Theory emphasizes that personal capabilities operate in interaction with environmental conditions (Bandura, 1986). In the present study, this environmental dimension is represented by perceived school ICT support, referring to students’ perceptions of the accessibility and availability of ICT-related resources and assistance within their learning environment. The broader literature describes school ICT support in terms of technological infrastructure, network resources, and technical guidance (Wang and Zhao, 2021). Because these conditions are measured through individual students’ self-reports in the present study, however, the construct reflects perceived access to school ICT support rather than an objective assessment of institutional infrastructure or service quality. This distinction is important because students with similar levels of AI Literacy may not experience the same opportunities to apply those capabilities when they perceive different levels of technological access and support.

Building on this reasoning, the present study develops and tests a moderated mediation model (Figure 1) to examine two related questions: whether Systematic Thinking Skills and cognitive load constitute cognitive pathways linking AI Literacy with self-reported Complex Problem-Solving Skills, and whether perceived school ICT support conditions the associations between AI Literacy and these cognitive factors. The study extends prior work in three respects. First, it examines these relationships in a higher vocational education population, where technology is closely connected with practice-oriented and industry-related learning. Second, it considers Systematic Thinking Skills and cognitive load simultaneously as complementary cognitive pathways rather than examining a single mechanism in isolation. Third, it incorporates perceived school ICT support as a student-level contextual moderator and temporally separates the focal predictor, cognitive factors, and outcome across three survey waves. In this way, the study does not seek to establish an entirely unknown association between AI Literacy and complex problem solving; rather, it examines how and under what perceived environmental conditions this relationship operates among vocational college students.

**Figure 1:**
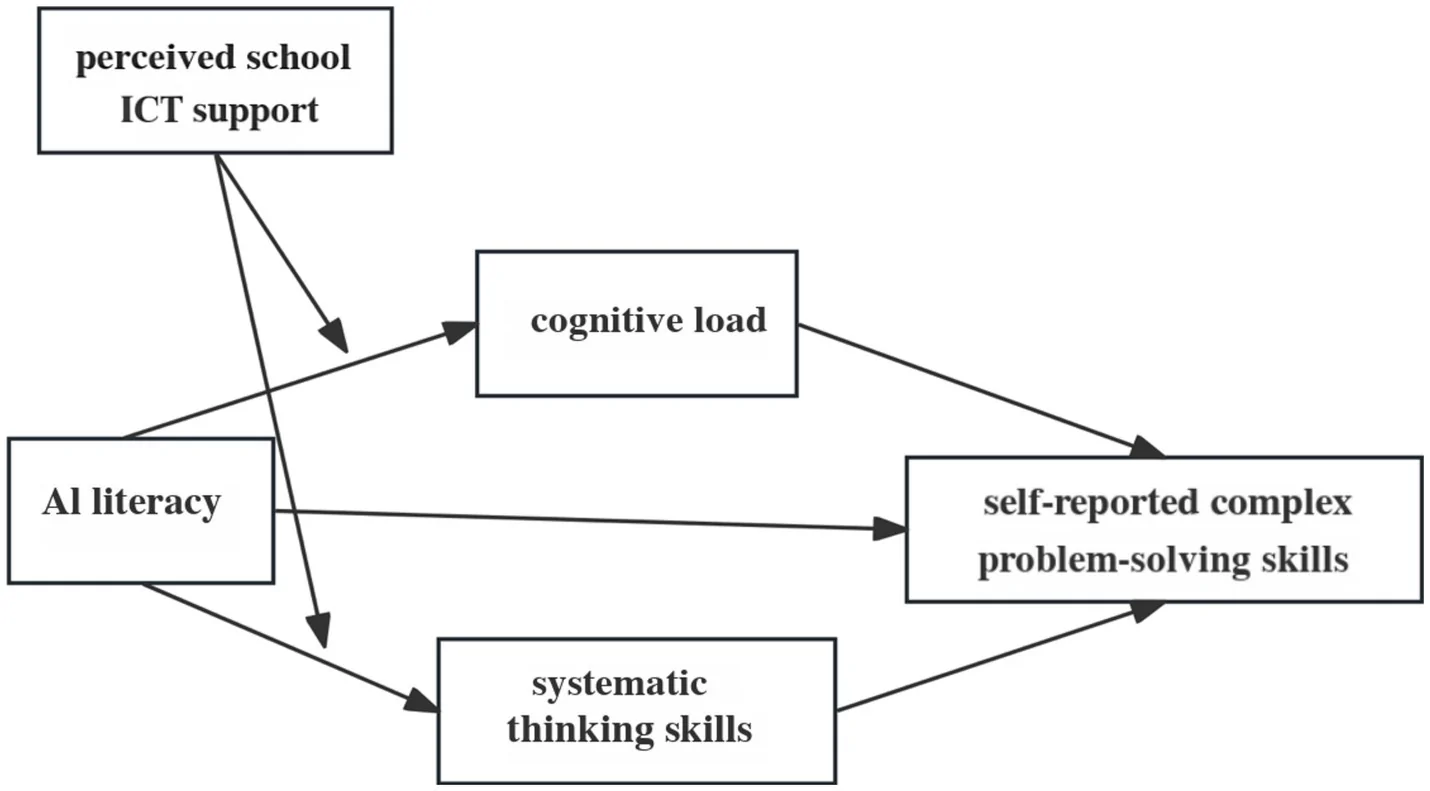
Conceptual model.

## 2 Literature review and hypothesis

### 2.1 The relationship between AI literacy and complex problem solving

Artificial Intelligence Literacy (AI Literacy) is defined as an individual’s comprehensive capability to effectively identify, apply, and evaluate artificial intelligence technologies and products (Long and Magerko, 2020; Wang et al., 2023). Specifically, it encompasses the entire process from recognizing fundamental AI concepts and mastering operational tools to critically evaluating AI outputs and making responsible ethical judgments (Perifanis and Kitsios, 2023; Sanusi et al., 2022). Within the context of higher vocational education, this literacy exhibits a strong practical orientation (Hazari, 2024; Chee et al., 2025). Recent evidence from higher vocational education further indicates that AI Literacy development can be organized around progressive cognitive understanding, practical skills application, and innovation-oriented competencies that are relevant to students’ preparation for AI-integrated occupational settings (Hong, 2025). It requires students not only to acquire proficiency in AI tools but also to apply them flexibly in authentic industry contexts, demonstrating both critical thinking and professional judgment. This integrated personal competency is regarded as a fundamental prerequisite for effectively addressing complex challenges.

According to Social Cognitive Theory, individual knowledge and skills represent personal capabilities that are relevant to how individuals approach challenging tasks (Abdullah, 2019). In the present study, AI Literacy is therefore conceptualized as a personal capability that may be positively associated with students’ self-reported Complex Problem-Solving Skills. Students with higher AI Literacy may be better able to extract relevant information from large amounts of data and critically evaluate the feasibility and limitations of AI-generated solutions in authentic contexts (Deranty and Corbin, 2024). These capabilities correspond closely to the information evaluation and judgment required when addressing complex problems. Previous research has also provided initial evidence linking AI Literacy with Complex Problem-Solving Skills and Systematic Thinking Skills (Promma et al., 2025). Related intervention-based evidence further suggests that structured AI Literacy education can strengthen learners’ ability to apply AI concepts to real-life problem solving and support metacognitive strategies during problem solving (Kong et al., 2024). However, evidence remains limited regarding how AI Literacy is associated with self-reported Complex Problem-Solving Skills among higher vocational students when additional cognitive and contextual factors are considered within the same model. Accordingly, we propose the following hypothesis:

*H1:* AI Literacy is positively associated with vocational college students’ self-reported Complex Problem-Solving Skills.

### 2.2 The mediating role of systematic thinking skills

In the present study, Systematic Thinking Skills refer to structured analytical processes involving problem structuring, reasoning, prioritization, and information integration, consistent with the operationalization used by Promma et al. (2025). Related systems-thinking literature provides broader conceptual support for the importance of identifying interrelationships, considering system dynamics, and approaching complex problems holistically (Hassan et al., 2020; York et al., 2019). Related empirical studies of systems thinking have also demonstrated links between holistic systems-oriented reasoning and complex problem solving (Melton et al., 2022; Grohs et al., 2018). Higher-education research likewise identifies systems thinking as an important capability for addressing complex problem-solving and decision-making situations and shows that such thinking can be developed through explicit systems-thinking methods (Elsawah et al., 2022). These skills enable individuals to analyze the internal structures and interconnections within complex problems. Complex problems often involve multiple interrelated and potentially conflicting factors (Dörner and Funke, 2017). Systematic Thinking Skills allow individuals to move beyond surface-level phenomena to identify the core components of a complex issue and to understand the dynamic causal relationships among these elements over time. Such analytical capacity facilitates the construction of an accurate and holistic mental representation of the problem (Schunk and DiBenedetto, 2020). At the organizational level, systems-oriented approaches have also been used to analyze nonlinear relationships and support learning and design processes in dynamic settings (Liboni et al., 2023). Furthermore, Systematic Thinking Skills encompass a prospective evaluative capacity, enabling individuals to use holistic representations to anticipate potential chain reactions when formulating solutions. This forward-looking reasoning supports the anticipation and assessment of subtle unintended consequences (Aujla et al., 2024), thereby preventing hasty decisions that address only immediate symptoms but may generate new problems in other parts of the system or in the future. Related systems-thinking approaches have been applied across diverse domains, including healthcare, ecological management, and agricultural operations (Brimble and Jones, 2017; Cummings et al., 2023; Hallett and Hobbs, 2020).

Previous studies have discussed AI Literacy in relation to learners’ understanding and use of AI in higher-education contexts (Alamäki et al., 2024), while more direct evidence linking AI Literacy with systematic thinking and complex problem solving has been reported by Promma et al. (2025). AI Literacy entails an understanding of AI as an input–process–output system. During interaction with AI tools, students are positioned not only as users but also as system analysts and debuggers. To obtain desired outcomes, causal links among data (input), algorithms (process), and generated content (output) are examined, and the system’s capability limits and potential biases are identified. Such interaction may involve core skills including decomposing systems, identifying elements and relations, and discerning overall system behavior (York et al., 2019).

According to Social Cognitive Theory, improvements in cognitive ability are derived from deep interactions between individuals and their environments (Abdullah, 2019). From this perspective, AI Literacy can be understood not only as tool use but also as a context in which systematic analysis may be practiced. Repeated engagement with the input–process–output logic of AI may encourage a more systematic approach to problem solving. Such a systematic approach may help students analyze complex problems from broader and more structured perspectives rather than relying primarily on trial-and-error. Evidence from an empirical study of accounting majors in Thai universities corroborates this view: systematic thinking was found to play a positive mediating role in the effect of AI Literacy on Complex Problem-Solving Skills (Promma et al., 2025). Accordingly, the following research hypothesis is proposed:

*H2a:* Systematic Thinking Skills mediate the relationship between AI Literacy and self-reported Complex Problem-Solving Skills.

### 2.3 The mediating role of cognitive load

Cognitive load refers to the cognitive demands placed on learners’ limited working-memory resources when they engage in a specific learning or problem-solving task (Seufert et al., 2024; Schneider et al., 2022). Cognitive Load Theory distinguishes among different sources and functions of cognitive load rather than treating all cognitive load as uniformly detrimental. In the framework underlying the instrument developed by Leppink et al. (2013), intrinsic load reflects the complexity of the learning content or task, extraneous load reflects demands arising from unclear or ineffective instructional features, and germane load reflects cognitive processing associated with developing understanding of the learning material. Because germane-load items reflect constructive learning-related processing rather than the task- and instruction-related cognitive demands examined in the present study, the four germane-load items were not included in the final analyses. The cognitive-load measure analyzed in this study therefore comprised three intrinsic-load items and three extraneous-load items. These dimensions represent distinct sources of cognitive demands, but both were considered relevant to the limited working-memory resources involved in complex problem solving.

Within AI-supported problem solving, AI Literacy may be relevant to students’ experienced cognitive load because familiarity with AI tools can influence how efficiently students interact with technological resources. Students with higher AI Literacy tend to have a clearer understanding of AI functions and expected outputs (Promma et al., 2025), which may reduce unnecessary cognitive demands associated with unfamiliar operations or information interference (Pinski and Benlian, 2024). Related evidence suggests that stronger digital skills can be associated with lower cognitive strain in educational settings, although this evidence comes from a different disciplinary context (Rehman et al., 2024). From a Social Cognitive Theory perspective, learning-related outcomes can be understood in relation to the interaction of personal and environmental factors (Bandura, 1986).

Cognitive load may, in turn, be related to complex problem solving because successful engagement with complex tasks requires learners to allocate limited cognitive resources across problem representation, information integration, reasoning, and decision-making. When task demands or unnecessary processing exceed available working-memory capacity, comprehension and reasoning may become less efficient (Sweller, 2024; Paas and Van Merriënboer, 2020). Excessive or extraneous demands can interfere with deeper processing and encourage reliance on less demanding strategies (Howard et al., 2020; Skulmowski et al., 2022; Akbulut et al., 2025). In complex problem-solving contexts, such demands may make it more difficult for learners to analyze problem structure, consider relationships among relevant factors, compare alternatives, and make well-supported decisions (Costley et al., 2024; Lange et al., 2023).

At the same time, the multidimensional nature of cognitive load requires caution in interpreting an overall cognitive-load score, because intrinsic, extraneous, and germane load represent theoretically distinct aspects of learners’ cognitive experience. Accordingly, the present study treats cognitive load as a theoretically relevant cognitive pathway, while the interpretation of its specific dimensions is tied to the measurement structure of the administered instrument. Therefore, the following hypothesis is proposed:

*H2b:* Cognitive load mediates the relationship between AI Literacy and self-reported Complex Problem-Solving Skills.

### 2.4 The moderating role of perceived school ICT support

School ICT support broadly refers to technological resources and assistance provided within educational institutions to help students and teachers use technology effectively, including infrastructure, software resources, training, and technical services (Nelson et al., 2019; Dong et al., 2020; Wang and Zhao, 2021). In vocational education, such support has been discussed as an important component of technology-supported learning environments (Duci et al., 2024).

In the present study, however, school ICT support is operationalized at the individual student level as perceived school ICT support. Specifically, the construct reflects students’ perceptions of how accessible ICT-related resources and assistance are within their school environment. It therefore does not represent an independently verified measure of institutional infrastructure, service quality, or school-level ICT capacity. This distinction is maintained throughout the theoretical development, hypothesis testing, and interpretation of the findings.

#### 2.4.1 The moderating role of perceived school ICT support in the relationship between AI literacy and systematic thinking skills

Based on social cognitive theory, high levels of environmental support can help learners more effectively leverage their abilities (Bandura, 1986). Therefore, this study posits that perceived school ICT support may condition the strength of the relationship between AI Literacy and Systematic Thinking Skills. On one hand, when students perceive AI-related tools, equipment, and technical assistance as readily accessible, they may have greater opportunities to apply what they have learned to authentic tasks (Shen and Cui, 2024; Jeilani and Abubakar, 2025). Students with higher AI Literacy possess the ability to understand problem structures and integrate information, but this ability requires a practical environment to be activated (Sanusi et al., 2022; Alamäki et al., 2024). Greater perceived access to ICT support may provide students with more opportunities to test their ideas and engage with AI-supported tasks (Walter, 2024; Shen and Cui, 2024), which may in turn encourage a more structured approach to understanding the overall problem, decomposing it into components, and refining its structure. In contrast, when perceived ICT support is lower, technology-related barriers may limit students’ opportunities to apply AI Literacy in systematic problem-solving activities.

On the other hand, sustained and stable technical support can reduce students’ frustration when using AI tools, creating a learning environment that encourages exploration and trial-and-error (Shen and Cui, 2024; Jeilani and Abubakar, 2025). Previous research suggests that supportive educational environments may be associated with students’ engagement and technology-related learning experiences (Sökmen, 2021; Jeilani and Abubakar, 2025). However, self-efficacy was not measured in the present study and is therefore treated as theoretical background rather than as a mechanism tested by the model. From a cognitive-load perspective, reducing unnecessary task demands can preserve limited working-memory resources for task-relevant processing (Paas and Van Merriënboer, 2020; Sweller et al., 2019). Thus, when students perceive ICT-related assistance as accessible, they may be better able to devote attention to the structure of the problem rather than to peripheral technological demands. This may allow them to focus more closely on problem structure and to apply systematic analytical processes more consistently. In contrast, in low ICT-support environments, the frustration caused by technical issues becomes a significant psychological barrier. This not only diminishes high AI-literacy students’ motivation to tackle complex tasks, but also leads them to remain in their comfort zone. Therefore, the following hypothesis is proposed:

*H3a:* Perceived school ICT support moderates the relationship between AI Literacy and Systematic Thinking Skills, such that the positive relationship is stronger when perceived school ICT support is high.

#### 2.4.2 The moderating role of perceived school ICT support in the relationship between AI literacy and cognitive load

Social cognitive theory posits that environmental resources assist learners in processing tasks more effectively, thereby influencing cognitive efficiency (Bandura, 1986). Accordingly, perceived school ICT support may moderate the strength of the relationship between AI Literacy and cognitive load. When perceived school ICT support is high, students report greater access to ICT-related resources and assistance. Such perceived accessibility may be associated with fewer technology-related barriers, including difficulties arising from unfamiliar interfaces, unstable access, or delayed technical assistance (Grinschgl and Neubauer, 2022; Chen and Chang, 2024). Timely access to support may also help students manage information and task demands more efficiently (Cai et al., 2024). Moreover, sufficient ICT support generally indicates that schools have access to adequate software resources. The automated features of these tools (such as intelligent analysis and data integration) may reduce some routine or unnecessary processing demands, leaving more working-memory resources available for task-relevant activities (Sajja et al., 2024). In such a comprehensive and convenient technological environment, students are less distracted by technical issues, allowing their limited working-memory capacity to be liberated from trivial technological concerns (Müller and Wulf, 2024). In other words, higher perceived ICT support may reduce some environmental constraints and may be associated with a stronger relationship between AI Literacy and cognitive load. In contrast, under low ICT support, the technological environment itself becomes a primary source of cognitive load, for example, through software lag, complex interfaces, or unstable network conditions. Students must expend significant working-memory resources to cope with these challenges, increasing task complexity, amplifying extraneous load, and diverting attention (Skulmowski et al., 2022). Therefore, the following hypothesis is proposed:

*H3b:* Perceived school ICT support moderates the relationship between AI Literacy and cognitive load, such that the negative relationship is stronger when perceived school ICT support is high.

Based on Social Cognitive Theory and Cognitive Load Theory, and building upon the preceding hypotheses, we propose that the indirect relationship between AI Literacy and self-reported Complex Problem-Solving Skills through these two pathways may vary across levels of perceived school ICT support and may be stronger when perceived school ICT support is high. Therefore, our next set of hypotheses posit a moderated mediation effect:

*H4a:* Perceived school ICT support moderates the indirect relationship between AI Literacy and self-reported Complex Problem-Solving Skills via Systematic Thinking Skills, such that the positive indirect relationship is stronger when perceived school ICT support is high.

*H4b:* Perceived school ICT support moderates the indirect relationship between AI Literacy and self-reported Complex Problem-Solving Skills via cognitive load, such that the positive indirect relationship is stronger when perceived school ICT support is high.

## 3 Methods

### 3.1 Sample and procedure

This study recruited students from six higher vocational colleges located in different areas of Guangdong Province, China. The participating institutions were purposively selected to reflect variation in the geographical distribution and ownership structure of higher vocational education in Guangdong Province. The sample included four public and two private vocational colleges, allowing the study to include students from different institutional contexts while maintaining institutional confidentiality. The matched analytic dataset retained school ownership (public or private), but did not contain anonymous college identifiers linking individual participants to the six participating institutions. Consequently, the numbers of matched participants from each college could not be reconstructed from the retained analytic dataset. Participants represented a range of disciplinary backgrounds within higher vocational education. Based on the academic-major categories recorded in the final analytic sample, 233 students (29.7%) were enrolled in humanities, history, philosophy, law, or education-related fields; 182 (23.2%) were enrolled in economics or management-related fields; 352 (44.9%) were enrolled in science, engineering, agriculture, or medicine-related fields; and 17 (2.2%) were enrolled in arts-related fields. The disciplinary diversity of the sample was retained in the analyses, and academic major was therefore treated as a nominal covariate rather than as an ordered continuous variable. Within each participating institution, the sampling frame consisted of first-, second-, and third-year students enrolled in the selected programmes who met the study eligibility criteria. Eligible participants were required to meet the following criteria: (a) current enrollment at one of the six participating higher vocational colleges; (b) enrollment as a first-, second-, or third-year student; (c) enrollment in one of the targeted programmes included in the study; (d) ability to complete the three-wave online questionnaires in Chinese; and (e) voluntary participation with informed consent.

Following institutional approval, faculty members and staff from student-affairs or academic-affairs offices at the participating colleges assisted the research team in contacting the relevant programmes and distributing the online survey invitation to eligible students. The questionnaire was administered through Wenjuanxing. Participation was entirely voluntary, and students reviewed the study information and informed-consent statement before deciding whether to participate. T1 data collection took place in March 2025, followed by T2 approximately four weeks later in April 2025 and T3 approximately four weeks after T2 in May 2025. No study-specific intervention was introduced between the three survey waves; participants continued their regular coursework and usual use of AI-related learning resources. The four-week interval was selected to provide temporal separation between measurements while maintaining continuity of participation across the three survey waves. At the initial time point (T1), a web link was sent to 920 voluntarily participating students, directing them to a designated web page. The web page provided detailed information about the research purpose and data collection process, ensuring anonymity and confidentiality. T1 assessed AI Literacy, perceived school ICT support, and demographic characteristics. After data screening, 878 valid T1 questionnaires were retained, representing 95.4% of the initial 920 cases. Approximately four weeks later, the 878 T1 participants were invited to complete T2, which assessed Systematic Thinking Skills and cognitive load. A total of 827 valid T2 responses were retained, corresponding to a T1-to-T2 retention rate of 94.2%. Approximately four weeks after T2, participants were invited to complete T3 and self-report their Complex Problem-Solving Skills. A total of 784 complete and matched cases were retained. This corresponded to a T2-to-T3 retention rate of 94.8% and a cumulative retention rate of 85.2% relative to the initial 920 cases. Table 1 summarizes participant retention and attrition across the three survey waves. Of the 920 initial cases, 784 were retained in the final matched analytic sample, while 136 were excluded during screening or not retained at subsequent waves. Because the individual-level baseline records for the nonretained cases and the original comparison outputs were unavailable, retained-versus-nonretained differences in T1 characteristics could not be reliably re-estimated or independently verified. Consequently, the possibility of selective attrition remains an important limitation of the study. In the final sample, 409 males (52.20%) were included (200 from urban households and 209 from rural households), and 375 females (47.80%) were included (142 from urban households and 233 from rural households). The sample consisted of 217 first-year students (27.70%), 251 s-year students (32.00%), and 316 third-year students (40.30%). All participants were 18 years of age or older; therefore, parental or guardian consent was not required. Responses across the three waves were matched using a participant-generated anonymous identification code that was entered at each survey occasion. The matching code was used solely for linking repeated responses and was stored separately from identifying information. The final analytic sample consisted of valid records successfully matched across T1, T2, and T3 using this anonymous identification code.

**Table 1:** Participant retention and attrition across three survey waves.

Survey stage	Timing	Number of cases	Reduction from the preceding stage
Initial T1 participation	March 2025	920	—
Valid T1 questionnaires	March 2025	878	42 excluded during initial screening
Valid T2 responses	April 2025	827	51 not retained after T1
Complete matched T3 cases	May 2025	784	43 not retained after T2

The final matched analytic sample comprised 784 of the 920 initially recorded cases, corresponding to a cumulative retention rate of 85.2%. Overall, 136 cases were not included in the final matched sample, comprising 42 records excluded during T1 screening, 51 cases not retained at T2, and 43 cases not retained at T3. Individual-level baseline records for the nonretained cases were unavailable for independent reanalysis. Consequently, retained-versus-nonretained baseline comparisons, statistical tests, and effect sizes could not be verified. The documented retention counts do not establish whether attrition was associated with measured or unmeasured participant characteristics.

### 3.2 Measures

Our focal variables were measured using well-established scales that have demonstrated sufficient reliability and validity. All scales used in this study were originally developed in English. We followed the standard translation and back-translation procedure (Brislin, 1970) to ensure linguistic and conceptual equivalence. To provide respondents with a common frame of reference, the questionnaire included a brief task-based response context before the focal measures. Participants were instructed to recall a recent complex learning, practical-training, project, or internship-related task that required problem identification, information integration, solution consideration, and task completion, potentially with the support of AI or other digital technologies. The task-based response context provided a reference for the systematic-thinking and cognitive-load measures at T2, whereas self-reported Complex Problem-Solving Skills were assessed at T3 using a perceived-capability scale. The available questionnaire documentation does not establish whether participants referred to the same specific task across the two waves or specify a fixed recall period. Accordingly, the association between cognitive load at T2 and self-reported problem-solving at T3 is not interpreted as a change in performance on an identical task. This contextualization was particularly important for the cognitive-load measure because cognitive load is theoretically task- and instruction-dependent rather than a context-free personal characteristic. Accordingly, the use of a concrete learning episode helped clarify the activity to which students’ perceived task complexity and instructional demands referred (Leppink et al., 2013). The items were first translated into Chinese by two professors specializing in vocational education. Subsequently, another professor from the same field, working independently, back-translated the Chinese version into English. A bilingual scholar then compared the original English version with the back-translated version. The two versions were found to be highly consistent, and any minor discrepancies were reconciled through discussion to finalize the Chinese version of the questionnaire. Unless otherwise noted, all items were rated on a 5-point Likert scale ranging from 1 (“Strongly Disagree”) to 5 (“Strongly Agree”). Scale scores were calculated as the mean of the corresponding items, with higher scores indicating higher levels of the measured construct.

#### 3.2.1 Complex problem-solving skills

Complex Problem-Solving Skills was measured using an eight-item self-report scale adapted from Promma et al. (2025), comprising four dimensions: Problem Identification (2 items, e.g., “You quickly identify key problems in complex scenarios”), Solution Generation (2 items, e.g., “You propose creative solutions to problems”), Iterative Testing (2 items, e.g., “You apply decision outcomes to improve performance”), and Stakeholder Analysis (2 items, e.g., “You manage activities to maximize benefits for all stakeholders”). Because the items ask students to evaluate their own problem-solving capabilities, the resulting scores represent students’ self-reported perceptions of CPS rather than objectively demonstrated performance on complex problem-solving tasks. The scale showed high internal consistency in the present study (Cronbach’s α = 0.928).

#### 3.2.2 Systematic thinking skills

Systematic Thinking Skills was measured using a scale adapted from Promma et al. (2025), comprising four dimensions: Structuring Problems (2 items, e.g., “You prioritize problems effectively before resolving them”), Reasoning (2 items, e.g., “You analyze facts, apply reasoning, and use principles to solve problems in complex situations”), Prioritizing (2 items, e.g., “You identify and address urgent and critical issues first to resolve problems efficiently.”), and Information Integration (2 items, e.g., “You consistently verify the credibility of various information sources before using them for decision-making.”). Accordingly, Systematic Thinking Skills in this study refer specifically to these four structured analytical dimensions and are not used interchangeably with the broader constructs of systems thinking or systemic thinking. In total, the scale comprises 8 items. The Cronbach’s alpha coefficient for this scale in the present study was 0.949.

#### 3.2.3 Cognitive load

Cognitive load was assessed using six items adapted from Leppink et al. (2013). The original 10-item instrument distinguishes intrinsic cognitive load (three items), extraneous cognitive load (three items), and germane cognitive load (four items). Because the hypothesized pathway in the present study concerns perceived cognitive demands that may interfere with complex problem solving, the four germane-load items were not included in the analyzed composite. Germane-load items capture constructive cognitive processing associated with learning and understanding and are therefore conceptually different from intrinsic task complexity and extraneous instructional demands.

Accordingly, the cognitive-load score used in the present analyses was calculated as the mean of six items representing intrinsic and extraneous cognitive load. The three intrinsic-load items assessed the perceived complexity of the referenced task, whereas the three extraneous-load items assessed cognitive demands associated with unclear or ineffective instructions or explanations. Although these dimensions represent distinct sources of cognitive load, both contribute to the cognitive demands experienced during learning and problem-solving activities (Kalyuga, 2011). The combined score was therefore used to summarize perceived task- and instruction-related cognitive demands within the referenced task, rather than to assume that intrinsic and extraneous load constitute a single underlying dimension. Participants responded with reference to the learning or practical task specified in the questionnaire’s response context. Higher scores indicated greater perceived cognitive demands captured by these six items. The six-item measure demonstrated acceptable internal consistency in the present sample (Cronbach’s α = 0.835). The intrinsic-load and extraneous-load subscales also demonstrated acceptable internal consistency (Cronbach’s α = 0.829 and 0.843, respectively).

#### 3.2.4 AI literacy

AI Literacy was measured using a scale adapted from Wang et al. (2023), comprising four dimensions: AI Awareness (e.g., “I can distinguish between smart devices and non-smart devices”), AI Use (e.g., “I am proficient at using AI applications or products to assist with my daily tasks”), AI Evaluation (e.g., “I can assess the capabilities and limitations of AI applications or products after using them for some time”), and AI Ethics (e.g., “I consistently adhere to ethical principles when using AI applications or products”). Each dimension was measured by three items, for a total of 12 items. The Cronbach’s alpha coefficient for this scale in the present study was 0.870.

#### 3.2.5 Perceived school ICT support

Perceived School ICT Support was measured using a three-item self-report scale from Wang and Zhao (2021), which was based on the work of Dong et al. (2020). The items assess students’ perceptions of the accessibility of ICT-related resources and support within their school environment, including support associated with ICT infrastructure, learning resources, and assistance from teachers, peers, or technical personnel. A sample item is, “I can obtain ICT-related support from my teachers or professional technicians easily.”

Because the items were completed by individual students, the resulting scores represent student-level perceptions of school ICT support rather than objective measures of institution-level infrastructure or support quality. Accordingly, the construct is referred to as perceived school ICT support throughout the manuscript. In the present study, the Cronbach’s alpha coefficient for the scale was 0.840.

#### 3.2.6 Control variables

To reduce potential confounding associated with individual and contextual differences, and consistent with prior research (Seker et al., 2025), several control variables were included in the regression-based analyses. These variables were gender (1 = male, 2 = female), household registration (1 = urban, 2 = rural), academic year (1 = first-year student, 2 = second-year student, 3 = third-year student), frequency of AI use (1 = less than once a month, 2 = at least once a month, 3 = more than once a week, 4 = once a day, 5 = multiple times a day), major category (1 = humanities, history, philosophy, law, or education, 2 = economics or management, 3 = science, engineering, agriculture, or medicine; 4 = arts), and school ownership (1 = public, 2 = private).

Because major category is nominal, it was dummy-coded for the regression-based analyses, with science, engineering, agriculture, and medicine (Category 3) serving as the reference group. Three dummy variables represented humanities/law/education (Category 1), economics/management (Category 2), and arts (Category 4), respectively. Academic year and AI-use frequency were entered as ordered trend covariates to adjust parsimoniously for progressively higher year levels and increasing frequency of AI use. Their coefficients were treated as adjustment terms reflecting an ordered linear trend across categories and were not substantively interpreted as focal effects.

School ownership and major category were included to account for potential systematic differences associated with institutional type and disciplinary background. Because class-level identifiers were not collected in accordance with the confidentiality requirements specified during ethical approval and institutional access, class-level clustering could not be modeled directly. Anonymous college identifiers were also unavailable in the retained analytic dataset, preventing the identification of college-level clusters. Although school ownership was included as a control variable, it does not account for potential dependence among students within the same college.

### 3.3 Data analysis methods

Confirmatory factor analysis and competing measurement-model comparisons were conducted using the full matched sample (N = 784), without selecting a separate CFA subsample. The same sample was used for the descriptive, correlation, and regression-based SPSS/PROCESS analyses. Data from the questionnaires were entered and analyzed using SPSS version 27.0 and Mplus 8.3. The analysis proceeded in three stages. First, potential common method variance was assessed using two complementary *post hoc* diagnostic approaches: Harman’s single-factor test, based on an unrotated principal components analysis of the 37 retained items, and an unmeasured latent method construct (ULMC) analysis. For Harman’s single-factor test, the 37 retained questionnaire items were examined using an unrotated principal components analysis, with particular attention to the proportion of total variance explained by the first component. The ULMC analysis was specified using the 37 original items, with equal loadings imposed on the common method factor. The ULMC model was evaluated in terms of both its global fit indices and the admissibility of its parameter estimates. Confirmatory factor analysis was conducted in Mplus to evaluate the measurement model and discriminant validity. The five-factor CFA used 25 observed indicators derived from the 37 retained questionnaire items. For AI Literacy, the original 12 items were summarized into four dimension-based mean parcels: AI Awareness (A_AWA, AISY1–AISY3), AI Use (A_USA, AISY4–AISY6), AI Evaluation (A_EVA, AISY7–AISY9), and AI Ethics (A_ETH, AISY10–AISY12). Similarly, the six cognitive-load items were represented by two dimension-based mean parcels: intrinsic load (C_IL, RZ1–RZ3) and extraneous load (C_EL, RZ4–RZ6). Each parcel was calculated as the mean of its three corresponding items. The eight Systematic Thinking Skills items (SW1–SW8), three perceived school ICT support items (ICT1–ICT3), and eight self-reported Complex Problem-Solving Skills items (SOLVE1–SOLVE8) were retained as individual indicators.

The CFA models were estimated using maximum likelihood (ML), with the 25 observed indicators treated as continuous variables, including the original five-point Likert items. Each indicator was specified to load on its corresponding latent factor, while the five factors were allowed to correlate. No cross-loadings or correlated indicator residuals were specified. The same 25-indicator structure was used for the competing measurement-model comparisons, with differences limited to the specified combinations of latent factors. By contrast, the six-item cognitive-load composite mean score was used for reliability estimation, descriptive statistics, correlation analyses, and regression-based mediation analyses. Thus, the same six cognitive-load items were retained across these analyses, although their representation differed between the CFA and the scale-score analyses. To examine the six-item cognitive-load measure without parceling, a separate item-level CFA compared a one-factor model (RZ1–RZ6 loading on one factor) with a correlated two-factor model (RZ1–RZ3 loading on intrinsic load and RZ4–RZ6 loading on extraneous load). Both models used the same six individual items and the full matched sample (N = 784).

Subsequently, descriptive statistics and correlation analyses were conducted for all variables. Finally, the mediation and moderated mediation hypotheses were tested using the PROCESS macro for SPSS, version 4.0 (Hayes et al., 2017). Parallel mediation was first examined using PROCESS Model 4, with Systematic Thinking Skills and cognitive load entered simultaneously as mediators. The moderated mediation model was then examined using PROCESS Model 7, with Systematic Thinking Skills and cognitive load entered simultaneously as parallel mediators and perceived school ICT support specified as the moderator of the associations between AI Literacy and each mediator. AI Literacy and perceived school ICT support were mean-centered before the interaction terms were constructed. Conditional indirect effects were estimated at the mean and at one standard deviation below and above the mean of perceived school ICT support. Statistical inference for indirect effects and indices of moderated mediation was based on 5,000 bootstrap samples and 95% percentile bootstrap confidence intervals. To examine the sensitivity of the cognitive-load findings to the scoring approach, the mediation and moderated mediation models were also estimated using intrinsic-load and extraneous-load scores as separate parallel mediators, alongside Systematic Thinking Skills. These sensitivity analyses used the same matched sample (N = 784) and 5,000 bootstrap samples as the primary analyses. The original PROCESS analyses used individual-level bootstrap resampling, with participant records rather than entire colleges serving as the sampling units. Because anonymous college identifiers were unavailable, the reported regression standard errors and bootstrap confidence intervals were not adjusted for potential dependence among students within the same college. An indirect or moderated mediation effect was considered statistically significant when the corresponding bootstrap confidence interval did not include zero. Simple-slope analyses were conducted to probe significant interaction effects. All regression-based analyses included the prespecified control variables: gender, household registration, academic year, frequency of AI use, school ownership, and the three dummy variables representing academic major.

## 4 Results

### 4.1 Descriptive statistics and correlation analysis

The descriptive statistics and Pearson correlation coefficients for all study variables are presented in Table 2 (N = 784). The mean scores for AI Literacy (*M* = 3.46, *SD* = 0.60), Systematic Thinking Skills (*M* = 3.70, *SD* = 0.67), cognitive load (*M* = 3.23, *SD* = 0.82), perceived school ICT support (*M* = 3.78, *SD* = 0.76), and self-reported Complex Problem-Solving Skills (*M* = 3.40, *SD* = 0.69) indicated moderate-to-high levels across the focal constructs.

**Table 2:** Means, standard deviations, and correlations among focal variables (*N* = 784).

Variables	*M*	SD	1	2	3	4	5	6	7	8	9	10	11
1. Gender	1.48	0.50	1										
2. Grade level	2.13	0.82	−0.067	1									
3. Household registration	1.56	0.50	0.111**	0.117**	1								
4. Academic major	2.20	0.89	−0.138**	0.254**	0.063	1							
5. School type	1.31	0.46	0.205**	−0.122**	0.063	−0.102**	1						
6. AI usage frequency	4.16	0.85	−0.014	0.035	0.068	−0.092*	0.057	1					
7. Self-reported Complex Problem-Solving Skills	3.40	0.69	−0.017	−0.169**	−0.106**	−0.120**	−0.114**	0.002	1				
8. AI Literacy	3.46	0.60	−0.014	0.057	−0.015	0.007	−0.102**	−0.031	0.421**	1			
9. Systematic Thinking Skills	3.70	0.67	−0.080*	0.046	−0.074*	0.023	−0.209**	−0.064	0.448**	0.471**	1		
10. Cognitive Load	3.23	0.82	0.033	−0.007	0.038	0.036	0.087*	0.012	−0.368**	−0.361**	−0.271**	1	
11. School ICT Support	3.78	0.76	0.041	−0.072*	−0.055	−0.044	−0.019	0.049	0.147**	0.055	0.315**	0.008	1

*N* = 784. M = mean; SD = standard deviation. Gender was coded as 1 = male and 2 = female; household registration as 1 = urban and 2 = rural; school ownership as 1 = public and 2 = private. *p < 0.05, **p < 0.01 (two-tailed). Academic major is a nominal variable; correlations involving its numeric category code are reported descriptively and are not interpreted substantively.

Bivariate correlations were generally consistent with the proposed relationships. AI Literacy was positively correlated with self-reported Complex Problem-Solving Skills (*r* = 0.421, *p* < 0.01) and Systematic Thinking Skills (*r* = 0.471, *p* < 0.01). Systematic Thinking Skills were also positively correlated with self-reported Complex Problem-Solving Skills (*r* = 0.448, *p* < 0.01). In contrast, cognitive load was negatively correlated with AI Literacy (*r* = −0.361, *p* < 0.01), self-reported Complex Problem-Solving Skills (*r* = −0.368, *p* < 0.01), and Systematic Thinking Skills (*r* = −0.271, *p* < 0.01). Perceived school ICT support was positively correlated with self-reported Complex Problem-Solving Skills (*r* = 0.147, *p* < 0.01) and Systematic Thinking Skills (*r* = 0.315, *p* < 0.01). None of the correlations among the focal constructs exceeded 0.70, suggesting that severe multicollinearity was unlikely.

### 4.2 Discriminant validity and common method Bias

Confirmatory factor analysis (CFA) was conducted using Mplus 8.3 with the final matched sample (N = 784) to evaluate the adequacy of the measurement model. As shown in Table 3, the hypothesized five-factor model demonstrated a good fit to the data: *χ*^2^ = 542.029, df = 265, *χ*^2^/df = 2.045, CFI = 0.977, TLI = 0.974, RMSEA = 0.037, and SRMR = 0.024. These fit indices were within commonly accepted ranges for adequate model fit (Hu and Bentler, 1999; Hair et al., 2010).

**Table 3:** Confirmatory factor analysis and discriminant validity.

Model	*χ* ^2^	*df*	*χ*^2^/*df*	RMSEA	CFI	TLI	SRMR
The hypothesized five-factor model: A, B, C, D, E	542.029	265	2.045	0.037	0.977	0.974	0.024
Four-factor model: A, B + E, C, D	3599.330	269	13.380	0.126	0.720	0.688	0.121
Four-factor model: A + B, C, D, E	975.449	269	3.626	0.058	0.941	0.934	0.057
Three-factor model a: A + B + C, D, E	1195.737	272	4.396	0.066	0.922	0.914	0.070
Two-factor model b: A + B + C + D, E	2036.431	274	7.432	0.091	0.852	0.838	0.087
One-factor model: A + B + C + D + E	4956.032	275	18.022	0.147	0.607	0.571	0.137

All competing measurement models were estimated using the final matched sample (*N* = 784) and the same 25 observed indicators. A = AI Literacy; B = Systematic Thinking Skills; C = Cognitive Load; D = Perceived School ICT Support; E = Self-reported Complex Problem-Solving Skills. AI Literacy was represented by four dimension-based mean parcels, and cognitive load by two dimension-based mean parcels. The remaining constructs were represented by their original items. All models used ML estimation.

To assess discriminant validity, the hypothesized five-factor model was compared with several alternative measurement models. The four-factor model in which systematic thinking skills and self-reported complex problem-solving skills were combined showed substantially poorer fit (*χ*^2^ = 3599.330, df = 269, *χ*^2^/df = 13.380, CFI = 0.720, TLI = 0.688, RMSEA = 0.126, SRMR = 0.121). The alternative four-factor model combining AI literacy and systematic thinking skills also showed poorer fit than the hypothesized model (*χ*^2^ = 975.449, df = 269, *χ*^2^/df = 3.626, CFI = 0.941, TLI = 0.934, RMSEA = 0.058, SRMR = 0.057). Similarly, the three-factor model showed poorer fit (*χ*^2^ = 1195.737, df = 272, *χ*^2^/df = 4.396, CFI = 0.922, TLI = 0.914, RMSEA = 0.066, SRMR = 0.070), as did the two-factor model (*χ*^2^ = 2036.431, df = 274, *χ*^2^/df = 7.432, CFI = 0.852, TLI = 0.838, RMSEA = 0.091, SRMR = 0.087). The one-factor model exhibited the poorest overall fit among the competing models (*χ*^2^ = 4956.032, df = 275, *χ*^2^/df = 18.022, CFI = 0.607, TLI = 0.571, RMSEA = 0.147, SRMR = 0.137). Taken together, these comparisons indicate that the hypothesized five-factor structure provided a better representation of the data than the alternative models and support the empirical distinctiveness of the five focal constructs. The standardized factor loadings in the hypothesized five-factor model ranged from 0.599 to 0.666 for AI literacy, 0.805 to 0.886 for systematic thinking skills, 0.636 to 0.716 for cognitive load, 0.779 to 0.813 for perceived school ICT support, and 0.720 to 0.811 for self-reported complex problem-solving skills. The standardized correlations among the five latent factors ranged from −0.529 to 0.564. These results provide further information about the measurement properties and relationships among the five constructs within the specified 25-indicator CFA model. An additional item-level CFA compared the one-factor and correlated two-factor representations of the six retained cognitive-load items. The one-factor model showed poor fit (*χ*^2^ = 865.906, df = 9, CFI = 0.547, TLI = 0.245, RMSEA = 0.348, SRMR = 0.116), whereas the correlated two-factor model fitted substantially better (*χ*^2^ = 24.207, df = 8, CFI = 0.991, TLI = 0.984, RMSEA = 0.051, SRMR = 0.024). The standardized correlation between intrinsic and extraneous load was 0.565. In the two-factor model, the standardized loadings were 0.842, 0.878, and 0.644 for RZ1–RZ3, respectively, and 0.807, 0.786, and 0.810 for RZ4–RZ6, respectively. These findings indicate that intrinsic and extraneous load are empirically distinguishable but related dimensions. Accordingly, the six-item composite used in the main mediation analyses is interpreted as a combined index of these cognitive demands, not as evidence that all six items measure one unidimensional latent factor. Potential common method variance was first examined using Harman’s single-factor test. An unrotated principal components analysis of the 37 retained questionnaire items was conducted using the final matched sample (N = 784). The first component accounted for 30.07% of the total variance. Although this result did not indicate that a single component accounted for most of the item variance, it was treated as a preliminary diagnostic rather than definitive evidence regarding the presence or absence of common method bias.

### 4.3 Results of the hypothesis tests

Table 4 summarizes the regression analyses. The reported coefficients are unstandardized regression coefficients (b), with standard errors presented in parentheses.

**Table 4:** Regression results.

Variable	Systematic Thinking Skills		Cognitive Load		Self-reported CPS
	Model 1–1	Model 1–2	Model 2–1	Model 2–2	Model 3
Controls					
Gender	−0.049 (0.043)	−0.061 (0.040)	0.038 (0.057)	0.015 (0.055)	0.009 (0.041)
Academic year	0.006 (0.027)	0.023 (0.025)	0.008 (0.035)	0.008 (0.034)	−0.151*** (0.026)
Household registration	−0.065 (0.043)	−0.053 (0.040)	0.033 (0.057)	0.053 (0.055)	−0.056 (0.041)
School ownership	−0.181*** (0.049)	−0.172*** (0.046)	0.093 (0.065)	0.075 (0.063)	−0.055 (0.048)
Frequency of AI use	−0.032 (0.025)	−0.042 (0.023)	−0.003 (0.033)	−0.011 (0.032)	0.032 (0.024)
Major 1: Humanities/history/philosophy/law/education	−0.038 (0.052)	−0.016 (0.049)	−0.110 (0.069)	−0.153* (0.067)	0.131** (0.050)
Major 2: Economics/management	0.034 (0.055)	0.031 (0.052)	−0.110 (0.073)	−0.153* (0.071)	0.153** (0.053)
Major 4: Arts	−0.308* (0.146)	−0.192 (0.137)	−0.312 (0.194)	−0.377* (0.187)	0.231 (0.141)
Predictors					
AI Literacy	0.499*** (0.034)	0.485*** (0.032)	−0.487*** (0.046)	−0.500*** (0.044)	0.248*** (0.039)
Mediators					
Systematic Thinking Skills	—	—	—	—	0.303*** (0.035)
Cognitive load	—	—	—	—	−0.166*** (0.026)
Moderator					
Perceived school ICT support	—	0.251*** (0.026)	—	0.035 (0.035)	—
AI Literacy × Perceived school ICT support	—	0.128*** (0.034)	—	−0.352*** (0.046)	—
*R* ^2^	0.260	0.353	0.140	0.202	0.352
Δ*R*^2^ for interaction	—	0.012	—	0.061	—
*F*	30.142***	38.321***	14.017***	17.729***	38.054***

*N* = 784. Entries are unstandardized regression coefficients (b), with standard errors in parentheses. All models included the same prespecified control variables: gender, academic year, household registration, frequency of AI use, school ownership, and academic major. Academic major was represented by three dummy variables, with science/engineering/agriculture/medicine (Category 3) serving as the reference group. Models 1–2 and 2–2 include the interaction between mean-centered AI Literacy and mean-centered perceived school ICT support. ΔR2 refers to the increase in explained variance attributable to the interaction term. *p < 0.05, **p < 0.01, ***p < 0.001. Coefficients are reported to three decimal places; identical displayed values may therefore reflect rounding of slightly different underlying estimates.

Hypothesis H1 proposed that AI Literacy would be positively associated with self-reported Complex Problem-Solving Skills. As shown in Table 5, the total effect of AI Literacy on self-reported Complex Problem-Solving Skills was positive and statistically significant (effect = 0.480, *SE* = 0.036, 95% CI [0.409, 0.550], *p* < 0.001). Therefore, H1 was supported.

**Table 5:** Results of the parallel mediation analysis for systematic thinking and cognitive load (bootstrapping = 5,000).

Effect Type	Effect	SE	LLCI	ULCI
Total effect	0.480	0.036	0.409	0.550
Direct effect	0.248	0.0390	0.172	0.324
Total indirect effect	0.232	0.028	0.181	0.288
AIL→STS → CPS	0.151	0.028	0.097	0.208
AIL→CL → CPS	0.081	0.018	0.049	0.119

AIL stands for “AI Literacy”; CL for “Cognitive Load,” STS for “Systematic Thinking Skills”, and CPS for “Complex Problem Solving Skills”.

Hypothesis H2a proposed an indirect relationship between AI Literacy and self-reported Complex Problem-Solving Skills through Systematic Thinking Skills. AI Literacy was positively associated with Systematic Thinking Skills (*b* = 0.499, *SE* = 0.034, *p* < 0.001). In the parallel mediation outcome model, Systematic Thinking Skills were positively associated with self-reported Complex Problem-Solving Skills (*b* = 0.303, *SE* = 0.035, *p* < 0.001), while the direct association between AI Literacy and self-reported Complex Problem-Solving Skills remained significant (*b* = 0.248, *SE* = 0.039, *p* < 0.001). Bootstrap analysis indicated a significant specific indirect effect through Systematic Thinking Skills (effect = 0.151, BootSE = 0.028, 95% CI [0.097, 0.208]). Therefore, H2a was supported.

Hypothesis H2b proposed an indirect relationship between AI Literacy and self-reported Complex Problem-Solving Skills through cognitive load. AI Literacy was negatively associated with cognitive load (*b* = −0.487, *SE* = 0.046, *p* < 0.001). In the parallel mediation outcome model, cognitive load was negatively associated with self-reported Complex Problem-Solving Skills (*b* = −0.166, *SE* = 0.026, *p* < 0.001), while the direct association between AI Literacy and self-reported Complex Problem-Solving Skills remained significant (*b* = 0.248, *SE* = 0.039, *p* < 0.001). Bootstrap analysis indicated a significant specific indirect effect through cognitive load (effect = 0.081, BootSE = 0.018, 95% CI [0.049, 0.119]). Therefore, H2b was supported.

To examine the two proposed indirect pathways simultaneously, a parallel mediation model was estimated. When both mediators were included, Systematic Thinking Skills (*b* = 0.303, *SE* = 0.035, *p* < 0.001) and cognitive load (*b* = −0.166, *SE* = 0.026, *p* < 0.001) were both significantly associated with self-reported Complex Problem-Solving Skills. The direct association between AI Literacy and self-reported Complex Problem-Solving Skills remained significant (*b* = 0.248, *SE* = 0.039, *p* < 0.001). As shown in Table 5, the total effect of AI Literacy on self-reported Complex Problem-Solving Skills was 0.480 (SE = 0.036, 95% CI [0.409, 0.550]), the direct effect was 0.248 (*SE* = 0.039, 95% CI [0.172, 0.324]), and the total indirect effect was 0.232 (BootSE = 0.028, 95% CI [0.181, 0.288]). The specific indirect effect through Systematic Thinking Skills was 0.151 (BootSE = 0.028, 95% CI [0.097, 0.208]), whereas the indirect effect through cognitive load was 0.081 (BootSE = 0.018, 95% CI [0.049, 0.119]). Because neither bootstrap confidence interval included zero, both indirect pathways were statistically significant.

Hypothesis H3a proposed that perceived school ICT support would moderate the positive association between AI Literacy and Systematic Thinking Skills. AI Literacy and perceived school ICT support were mean-centered before constructing the interaction term. As shown in Table 4 (Model 1–2), the interaction between AI Literacy and perceived school ICT support was positive and statistically significant (*b* = 0.128, *SE* = 0.034, *p* < 0.001). Simple-slope analysis indicated that the positive association between AI Literacy and Systematic Thinking Skills was significant at a low level of perceived school ICT support (*M* − 1 SD; simple slope = 0.387, *t* = 9.549, *p* < 0.001) and became stronger at a high level of perceived school ICT support (*M* + 1 SD; simple slope = 0.582, *t* = 13.913, *p* < 0.001). Therefore, H3a was supported (Figure 2).

**Figure 2:**
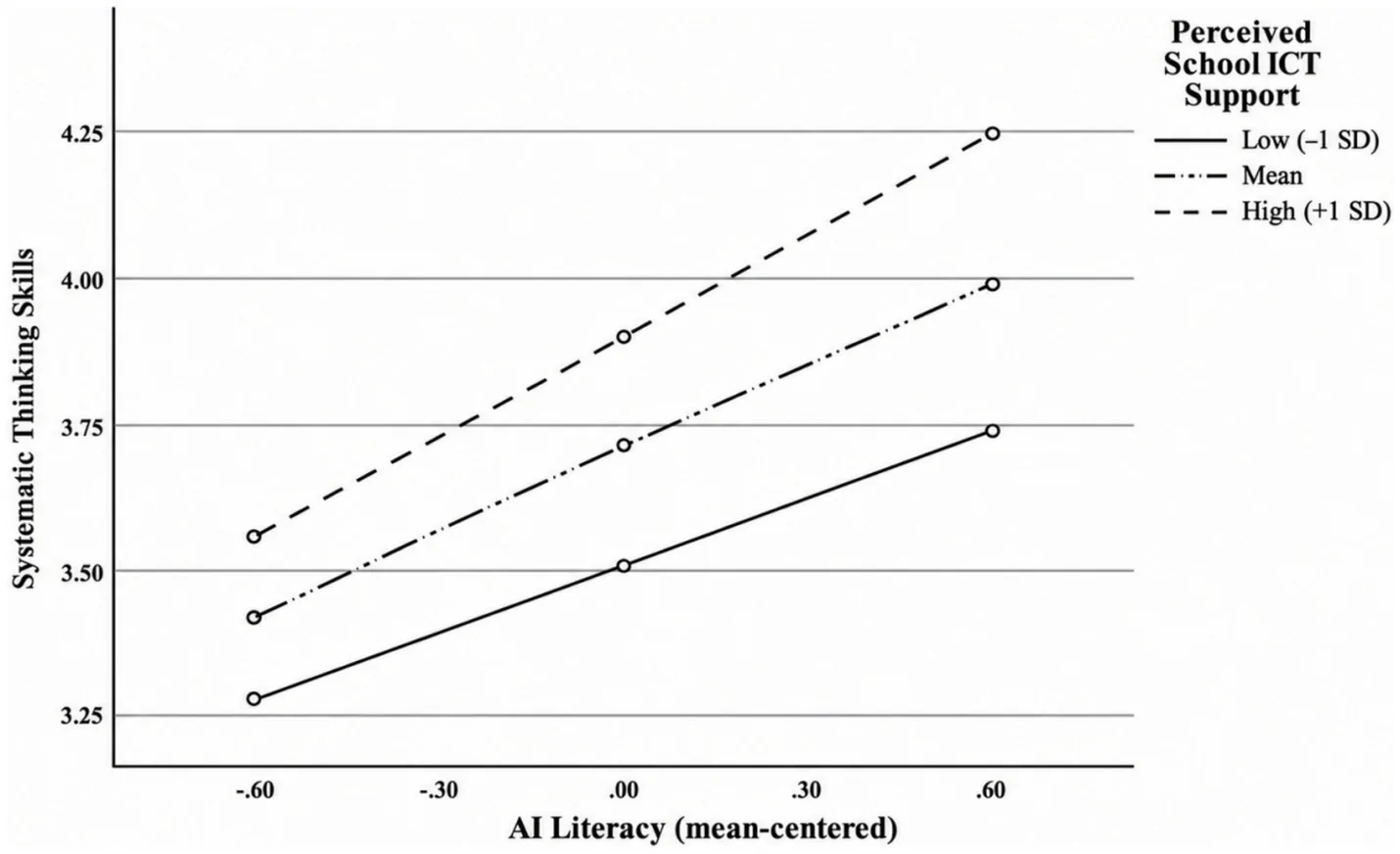
Moderating effect of perceived school ICT support on the relationship between AI literacy and systematic thinking skills.

Hypothesis H3b proposed that perceived school ICT support would moderate the relationship between AI Literacy and cognitive load. As shown in Table 4 (Model 2–2), the interaction between AI Literacy and perceived school ICT support was negative and statistically significant (*b* = −0.352, *SE* = 0.046, *p* < 0.001). Simple-slope analysis showed that AI Literacy was negatively associated with cognitive load when perceived school ICT support was low (*M* − 1 SD; simple slope = −0.231, *t* = −4.154, *p* < 0.001), and this negative association became stronger when perceived school ICT support was high (*M* + 1 SD; simple slope = −0.769, *t* = −13.394, *p* < 0.001). Therefore, H3b was supported (Figure 3).

**Figure 3:**
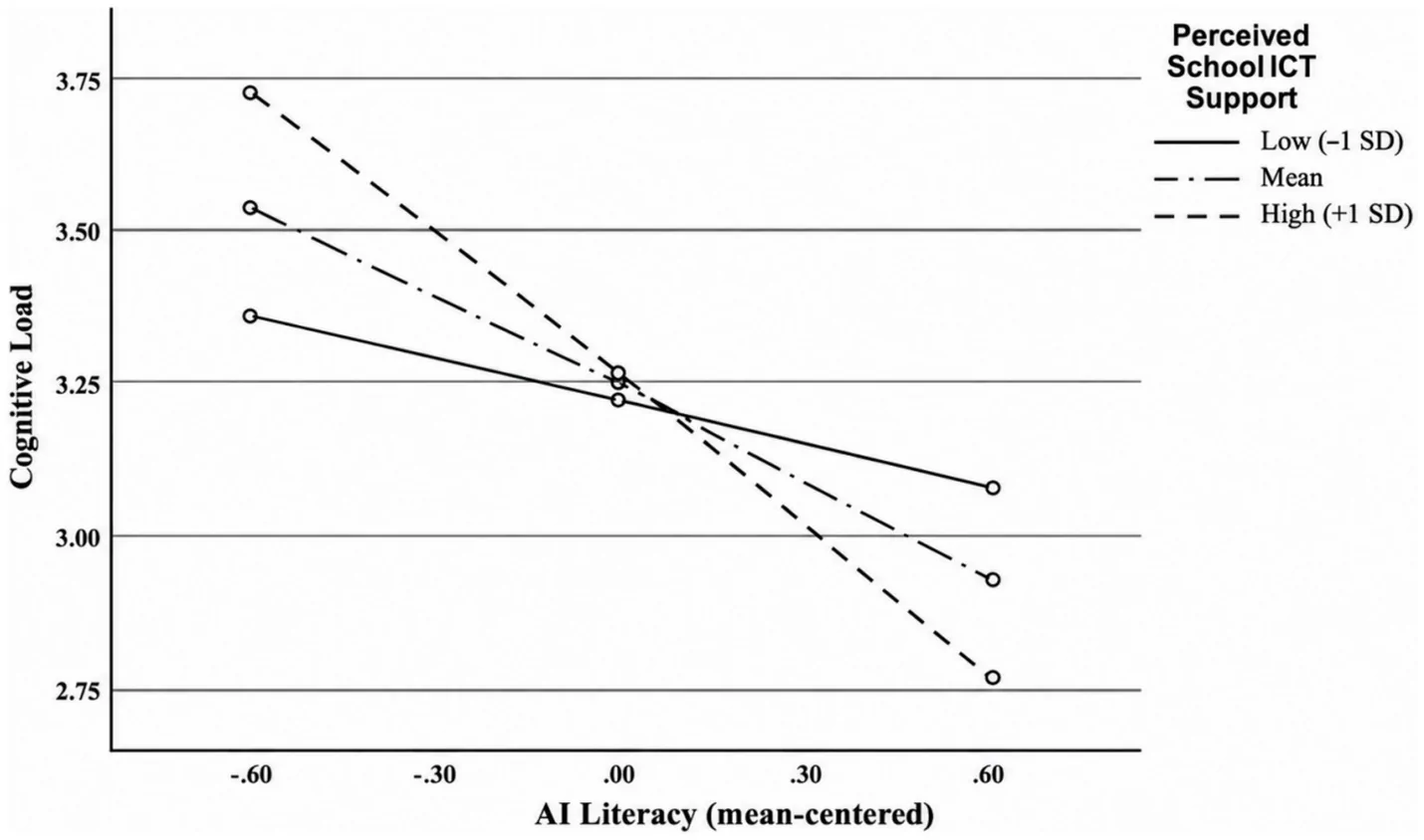
Moderating effect of perceived school ICT support on the relationship between AI literacy and cognitive load.

Hypothesis H4a proposed that the indirect relationship between AI Literacy and self-reported Complex Problem-Solving Skills through Systematic Thinking Skills would vary across levels of perceived school ICT support. As shown in Table 6, the conditional indirect effect through Systematic Thinking Skills was significant at a low level of perceived school ICT support (*M* − 1 SD = −0.764; effect = 0.117, BootSE = 0.032, 95% CI [0.053, 0.178]), at the mean level of perceived school ICT support (effect = 0.147, BootSE = 0.027, 95% CI [0.096, 0.200]), and at a high level of perceived school ICT support (*M* + 1 SD = 0.764; effect = 0.176, BootSE = 0.030, 95% CI [0.122, 0.240]). The index of moderated mediation was significant (index = 0.039, BootSE = 0.021, 95% CI [0.008, 0.089]). Therefore, H4a was supported.

**Table 6:** Conditional indirect effects (bootstrapping = 5,000).

Mediator	Moderator	Effect	SE	95% CI
Systematic thinking skills (M1)	Low (−1 SD, −0.764)	0.117	0.032	[0.053, 0.178]
	Medium (Mean, 0.000)	0.147	0.027	[0.096, 0.200]
	High (+1 SD, +0.764)	0.176	0.030	[0.122, 0.240]
	Index of Moderated Mediation	0.039	0.021	[0.008, 0.089]
Cognitive load (M2)	Low (−1 SD, −0.764)	0.038	0.012	[0.016, 0.064]
	Medium (Mean, 0.000)	0.083	0.017	[0.052, 0.117]
	High (+1 SD, +0.764)	0.127	0.025	[0.082, 0.178]
	Index of Moderated Mediation	0.058	0.013	[0.035, 0.089]

Hypothesis H4b proposed that the indirect relationship between AI Literacy and self-reported Complex Problem-Solving Skills through cognitive load would vary across levels of perceived school ICT support. The conditional indirect effect through cognitive load was significant at a low level of perceived school ICT support (*M* − 1 *SD* = −0.764; effect = 0.038, BootSE = 0.012, 95% CI [0.016, 0.064]), at the mean level of perceived school ICT support (effect = 0.083, BootSE = 0.017, 95% CI [0.052, 0.117]), and at a high level of perceived school ICT support (*M* + 1 *SD* = 0.764; effect = 0.127, BootSE = 0.025, 95% CI [0.082, 0.178]). The index of moderated mediation was also significant (index = 0.058, BootSE = 0.013, 95% CI [0.035, 0.089]). Therefore, H4b was supported. Sensitivity analyses were conducted by entering intrinsic and extraneous cognitive load as separate parallel mediators, alongside Systematic Thinking Skills. In PROCESS Model 4, the indirect effects through intrinsic load (effect = 0.0487, BootSE = 0.0142, 95% CI [0.0232, 0.0785]) and extraneous load (effect = 0.0323, BootSE = 0.0132, 95% CI [0.0071, 0.0591]) were both positive, with neither confidence interval including zero. The contrast between these indirect effects was not statistically significant (effect = 0.0164, 95% CI [−0.0233, 0.0576]). In PROCESS Model 7, the indices of moderated mediation were also positive for intrinsic load (index = 0.0357, BootSE = 0.0105, 95% CI [0.0170, 0.0586]) and extraneous load (index = 0.0236, BootSE = 0.0101, 95% CI [0.0063, 0.0463]). These results were consistent in direction with the primary composite-score analyses, while indicating that both dimensions showed indirect associations when examined separately within the same model. These results were obtained from individual-level regression analyses and bootstrap procedures without adjustment for potential within-college dependence. Accordingly, the statistical significance of the reported associations and indirect effects should be interpreted cautiously, as their uncertainty may be underestimated if responses from students within the same college are correlated.

## 5 Discussion

This study, based on Social Cognitive Theory, focuses on vocational college students and examines the relationships among AI Literacy, self-reported Complex Problem-Solving Skills, Systematic Thinking Skills, cognitive load, and perceived school ICT support through a three-wave questionnaire survey. The findings indicate that (1) AI Literacy was significantly and positively associated with self-reported Complex Problem-Solving Skills; (2) Systematic Thinking Skills and cognitive load were involved in significant indirect relationships between AI Literacy and self-reported Complex Problem-Solving Skills; and (3) the associations between AI Literacy and the two cognitive factors varied across levels of perceived school ICT support. These findings should be interpreted as associations among time-separated self-report measures rather than as evidence that AI Literacy produces changes in cognitive processes or objectively demonstrated problem-solving performance.

### 5.1 Theoretical implications

First, the findings showed that AI Literacy was positively associated with vocational college students’ self-reported Complex Problem-Solving Skills. This result is consistent with Promma et al. (2025), who also reported a positive relationship between AI Literacy and Complex Problem-Solving Skills, while the present study extends this association to Chinese higher vocational education. More recent evidence likewise suggests that AI Literacy is associated with students’ use of AI for learning and that such engagement is related to critical-thinking and problem-solving skills (Sultana et al., 2026). One explanation is that AI Literacy involves more than familiarity with AI applications. Students with stronger AI Literacy are better positioned to identify when AI may be useful, evaluate generated information, recognize the limitations of AI outputs, and integrate such information into their own judgment rather than relying on generated responses uncritically (Long and Magerko, 2020; Wang et al., 2023). These capabilities are particularly relevant to complex problems involving incomplete information, interacting variables, multiple constraints, and changing decision conditions (Dörner and Funke, 2017).

This relationship has particular relevance to higher vocational education, where students are prepared for practice-oriented and increasingly technology-rich occupational contexts (Chee et al., 2025). For students in technical programmes, for example, debugging a program may involve using AI to identify possible sources of an error while still judging which suggestion is technically appropriate and adapting it to actual operating conditions. In such situations, AI Literacy supports not simply access to AI-generated information, but the purposeful integration of that information with students’ own technical judgment. From a Social Cognitive Theory perspective, AI Literacy can therefore be understood as a personal capability associated with how students approach complex tasks. The contribution of this finding lies in showing that the AI Literacy–problem-solving relationship is also evident in a practice-oriented Chinese vocational context. This extension is relevant to broader higher-education research emphasizing transferable meta-skills in preparing students for changing professional demands (Che et al., 2026a).

Second, systematic thinking constituted a significant indirect pathway linking AI Literacy with self-reported Complex Problem-Solving Skills. Students with higher AI Literacy reported stronger Systematic Thinking Skills, which were in turn associated with higher perceived complex problem-solving capability. This finding is consistent with Promma et al. (2025), who identified a similar mediating relationship among Thai accounting students, while the present study extends evidence for this pathway to Chinese higher vocational education.

The first part of this pathway may be explained by the cognitive activities involved in effective interaction with AI. Productive AI use rarely ends with accepting the first generated response. Students often need to clarify a problem, specify relevant conditions, compare alternative outputs, identify inconsistencies, integrate information from different sources, and revise their queries or decisions. These activities require problem structuring, reasoning, prioritization, and information integration, which closely correspond to the Systematic Thinking Skills examined in this study. Recent work on discipline-specific AI Literacy similarly emphasizes problem articulation, disciplinary reasoning, and evaluation of the consequences of AI-supported decisions (Stolpe et al., 2026). This interpretation is also consistent with systems-thinking research emphasizing relationships among multiple elements (York et al., 2019) and with work showing that meaningful AI engagement involves understanding and evaluating AI rather than using it only instrumentally (Alamäki et al., 2024). Related research on GenAI-supported learning further suggests that AI-generated materials can provide opportunities for systems-oriented analysis when generated relationships and assumptions are critically evaluated (Courchesne et al., 2026).

The second part of the pathway concerns why systematic thinking is associated with complex problem solving. Complex problems typically involve incomplete information, interacting variables, competing constraints, and consequences that change as decisions unfold (Dörner and Funke, 2017). Systematic thinking can help students form a more structured representation of these relationships, identify central elements, distinguish important constraints from less relevant information, and compare alternative courses of action rather than relying on isolated trial and error. This reasoning is consistent with research linking systems-oriented analysis with ill-structured problem solving (Grohs et al., 2018) and with recent work emphasizing systems thinking in professional decisions involving feedback, uncertainty, and unintended consequences (Pedraja-Rejas et al., 2026). In Chinese higher vocational education, this process may be especially useful in technical tasks involving several procedures, information sources, and constraints. When diagnosing a technical fault, for example, a student may need to distinguish relevant symptoms, examine possible causes, compare AI-generated suggestions with technical knowledge, and determine which solution fits the actual task conditions. AI Literacy helps the student use and evaluate AI-generated information, whereas systematic thinking helps organize that information into a coherent problem-solving process. The present findings therefore add to prior work by identifying systematic thinking as a cognitive pathway through which AI-related capability is associated with perceived complex problem-solving capability in a practice-oriented vocational context.

Third, cognitive load, as represented by the six-item composite of intrinsic and extraneous cognitive demands, constituted a second significant indirect pathway between AI Literacy and self-reported Complex Problem-Solving Skills. The separate-score sensitivity analyses further showed positive indirect associations through both intrinsic and extraneous load, although the item-level CFA indicated that these dimensions were empirically distinguishable. Higher AI Literacy was associated with lower cognitive-load scores, which were in turn associated with higher perceived complex problem-solving capability. This pattern is consistent with evidence linking stronger digital skills with lower cognitive strain in educational settings (Rehman et al., 2024). Research also shows that greater task or instructional difficulty can increase cognitive load and reduce operational efficiency (Eesee et al., 2025), highlighting the importance of how learners manage cognitive demands during complex activities.

One explanation for the negative association between AI Literacy and cognitive load is that students with greater AI Literacy have clearer expectations about what AI systems can and cannot do, how prompts or instructions should be formulated, and how generated information should be evaluated. This familiarity may reduce extraneous demands arising from inefficient tool use, repeated searches, poorly formulated prompts, or attention to irrelevant outputs. AI Literacy may also help students manage cognitively demanding task requirements by identifying relevant information, organizing inputs, and using AI resources selectively rather than attempting to process all available information simultaneously. This does not imply that AI Literacy changes the objective complexity of a task; rather, it may make the cognitive demands of working through that complexity more manageable. This interpretation is consistent with research on purposeful and efficient engagement with AI systems (Pinski and Benlian, 2024), the relationship between AI-supported learning environments and cognitive load (Koć-Januchta et al., 2022), and recent evidence that structured and transparent GenAI support can be associated with lower perceived cognitive load (Jou and Hao, 2026).

Recent findings also suggest that AI-related cognitive demands should not be understood as uniformly decreasing with greater AI use. Zhang and Jiang (2026), for example, reported that extraneous load was lowest at moderate levels of GenAI dependency and that higher-order thinking also varied with the degree of dependency. Their study concerns AI dependency rather than AI Literacy, so the findings are not directly equivalent to those reported here. They nevertheless highlight an important distinction: effective AI capability may reduce inefficient processing, whereas excessive reliance on AI may introduce a different set of cognitive consequences.

Lower scores on the six-item cognitive-load composite were associated with higher self-reported Complex Problem-Solving Skills. One possible explanation is that manageable task demands and reduced unnecessary processing may allow students to allocate more of their limited working-memory resources to problem representation, information integration, reasoning, comparison of alternatives, and decision-making. This explanation is consistent with Cognitive Load Theory (Paas and Van Merriënboer, 2020; Sweller, 2024) and with evidence that excessive cognitive demands can undermine deeper processing and problem solving (Costley et al., 2024; Lange et al., 2023). This mechanism is relevant to Chinese higher vocational education, where practical tasks may require students to interpret technical information, operate digital tools, evaluate AI-generated suggestions, and consider task-specific constraints at the same time.

Taken together with the systematic-thinking pathway, the two indirect relationships point to complementary cognitive processes. AI Literacy was associated with how students organized and analysed complex information through Systematic Thinking Skills, as well as with lower scores on the six-item composite of intrinsic and extraneous cognitive demands. Thus, the model provides a more differentiated explanation of the AI Literacy–problem-solving relationship than a single direct pathway alone. This interpretation should nevertheless remain within the boundaries of the cognitive-load measure used in this study. Because the final measure included intrinsic- and extraneous-load items but not germane-load items, the observed relationship should be understood as applying to the cognitive demands captured by these retained items rather than to cognitive load in all of its forms.

Finally, perceived school ICT support functioned as a contextual condition for both cognitive pathways. The positive association between AI literacy and systematic thinking was stronger at higher levels of perceived ICT support, and the negative association between AI literacy and cognitive load also became stronger as perceived support increased. From a social cognitive theory perspective, these findings are consistent with the view that personal capabilities operate in interaction with environmental conditions rather than independently of them. In the present study, AI literacy represents a personal capability, whereas perceived school ICT support reflects students’ experience of the technological resources and assistance available in their learning environment.

Higher perceived ICT support may strengthen the AI literacy–systematic thinking relationship by giving students greater opportunities to use AI-supported tools, test ideas, compare outputs, and obtain assistance when technical difficulties arise. Under these conditions, students with stronger AI literacy are better able to put their existing capability into practice through repeated problem structuring, information comparison, and evaluation. The same environmental support can also help explain the stronger negative association between AI literacy and cognitive load. Accessible resources and timely assistance may reduce demands arising from access problems, unstable platforms, unfamiliar interfaces, or inefficient tool use, allowing students’ existing AI capability to be applied with less peripheral cognitive effort. When such support is limited, these environmental demands can consume attention and reduce opportunities to use AI literacy effectively.

This interpretation is consistent with previous research linking technical and educational support with students’ AI or digital capabilities and technology-related learning experiences (Shen and Cui, 2024; Chiu et al., 2022; Wang and Zhao, 2021), as well as research emphasizing the role of external technological resources in cognitive activity (Grinschgl and Neubauer, 2022). Recent evidence from Chinese higher education similarly associates broader institutional support for GenAI with students’ perceived capability development across different patterns of GenAI use (Zhang et al., 2026). The present study differs from this earlier work in an important respect: perceived ICT support was not examined only as a direct predictor of technology use or capability development, but as a condition associated with the strength of two specific cognitive pathways. For Chinese higher vocational education, this suggests that students’ AI-related capability and their perceived technological environment should be considered jointly. The moderated mediation results therefore identify a contextual boundary to the relationship between AI literacy and self-reported complex problem-solving capability: similar levels of AI literacy may be associated with different cognitive processes depending on the ICT support students perceive around them.

### 5.2 Practical implications

This study examines how AI Literacy is related to self-reported Complex Problem-Solving Skills among vocational college students from the perspective of Social Cognitive Theory and discusses potential educational applications based on the observed relationships.

First, vocational colleges may consider integrating systematic AI Literacy learning opportunities into their curricula. This study found that higher AI Literacy was associated with higher self-reported Complex Problem-Solving Skills, with additional indirect associations through the proposed cognitive pathways. This finding aligns with the growing consensus that AI Literacy has become increasingly important for individuals’ effective participation in the digital age (Zhang et al., 2023). Accordingly, educators and policymakers may consider incorporating AI Literacy into curricula and teaching strategies while further evaluating its educational effects in future intervention-based research. One possible application for future evaluation is a modular AI Literacy course that combines foundational AI concepts, ethical considerations, and discipline-relevant use of AI tools.

Moreover, vocational colleges may consider embedding opportunities to practice Systematic Thinking Skills within AI-related courses. From the perspective of Social Cognitive Theory, the findings are consistent with the proposed mediating role of Systematic Thinking Skills in the relationship between AI Literacy and self-reported Complex Problem-Solving Skills. Because the systematic thinking construct examined in this study comprises problem structuring, reasoning, prioritization, and information integration, instructional activities could be designed to provide students with opportunities to practice these processes when working with AI-supported tasks. For example, students could first structure an industry-related problem, compare alternative AI-generated outputs using explicit reasoning, identify and prioritize relevant constraints, and integrate information from multiple sources before reaching a decision. Such activities would align more closely with the specific Systematic Thinking Skills examined in this study rather than treating the construct as a broad form of systems thinking. These potential applications may be more meaningful when considered as part of coordinated educational design rather than as isolated instructional activities. Recent higher-education research on meta-skills similarly emphasizes the alignment of curriculum, pedagogy, and assessment and the use of design-oriented approaches to translate broad capability priorities into concrete learning arrangements (Che, 2026). Related evidence from Chinese higher education further suggests that meta-skills development should be considered within broader academic-management arrangements rather than as an isolated learner-level objective (Che et al., 2026b). From this perspective, opportunities to develop AI Literacy and practice systematic thinking could be incorporated across learning tasks, instructional guidance, and assessment activities, while their effectiveness should be evaluated empirically rather than assumed from the present findings.

Finally, vocational colleges may consider how accessible and usable students perceive ICT-related resources and support to be. The present findings indicate that higher perceived school ICT support was associated with variation in the relationships between AI Literacy, Systematic Thinking Skills, and cognitive load. Because the measure captures students’ perceptions rather than objective institution-level ICT quality, the findings should not be interpreted as direct evidence that particular infrastructure investments or support arrangements will produce better cognitive outcomes.

Nevertheless, the results suggest that institutions may benefit from evaluating whether students can readily access the technological resources, guidance, and technical assistance needed for AI-supported learning. Potential directions for future evaluation include targeted investments in relevant software and computing resources, piloting dedicated IT or educational technology support teams, and ensuring that students are aware of available ICT support services. Where appropriate, institutions could also test initiatives such as AI innovation funds or interdisciplinary human–machine collaboration competitions to examine whether they provide meaningful opportunities for students to apply AI-related knowledge in authentic problem-solving activities. These options should be treated as interventions warranting future evaluation rather than as practices validated by the present study.

### 5.3 Limitations and future research

This study has several limitations. First, the sample was drawn from six higher vocational colleges in Guangdong Province, which may limit the generalizability of the findings to vocational students in other regions, institutional settings, or disciplinary contexts. Although participant retention across the three survey waves was documented, the individual-level baseline records for nonretained participants and the original attrition-comparison outputs were unavailable for independent verification. Therefore, we could not reliably determine whether retention was associated with participants’ measured baseline characteristics, and the possibility of selective attrition cannot be excluded. Future studies could examine the model using more geographically and academically diverse samples and retain the baseline information needed to evaluate attrition-related differences.

Second, although the focal variables were temporally separated across three survey waves, each construct was assessed only once and baseline levels of the mediators and outcome were not measured. The study could not verify whether responses at T2 and T3 referred to the same specific task. The observed indirect relationships therefore cannot establish within-person change, developmental processes, or causality. Future studies could use repeated-measures longitudinal or experimental designs to examine these relationships more directly.

Third, all focal variables were self-reported, and the CPS measure reflects students’ perceived problem-solving capability rather than objectively demonstrated performance. Although the three-wave design provided temporal separation, the measures were completed by the same respondents, and shared-method bias cannot be excluded. Moreover, Harman’s single-factor test and the ULMC analysis were used as *post hoc* diagnostics, neither of which could establish the absence of common method bias. Future research could combine self-reports with performance-based tasks, simulations, or independently assessed indicators.

Fourth, students were nested within six colleges, but class-level identifiers and anonymous college identifiers were unavailable in the retained analytic dataset, preventing the direct assessment of clustering. Although school ownership was included as a control variable, it does not account for potential dependence among students within the same college. Consequently, the reported regression standard errors and individual-level bootstrap confidence intervals may underestimate statistical uncertainty if within-college correlations are present. The statistical significance of the observed associations and indirect effects should therefore be interpreted cautiously. Future studies with identifiable class or institutional structures and a larger number of participating colleges could use multilevel designs and appropriate cluster-adjusted inference to examine whether these relationships vary across educational contexts.

Fifth, the analyzed cognitive-load score combined retained intrinsic- and extraneous-load items, while germane-load items were excluded because they represent constructive learning-related processing. Although the separate-score sensitivity analyses showed positive indirect associations through both dimensions, the item-level CFA supported treating intrinsic and extraneous load as distinct but related constructs. The findings therefore apply to this six-item cognitive-load measure and should not be generalized to all dimensions of cognitive load. Future research could further examine the distinct roles of intrinsic and extraneous load across different learning contexts and assess germane processing as a separate construct.

## 6 Conclusion

This study, grounded in Social Cognitive Theory, examined an integrated model of the relationships among AI Literacy, self-reported Complex Problem-Solving Skills, Systematic Thinking Skills, cognitive load, and perceived school ICT support among vocational college students. The results showed that higher AI Literacy was associated with higher self-reported Complex Problem-Solving Skills, with significant indirect associations estimated through Systematic Thinking Skills and the six-item cognitive-load composite. Perceived school ICT support also emerged as a contextual condition associated with variation in the relationships between AI Literacy and the two cognitive factors. Taken together, these findings are consistent with the proposed view that individual AI Literacy, cognitive processes, and students’ perceptions of environmental support are interrelated with vocational students’ perceptions of their complex problem-solving capabilities. Theoretically, the study provides evidence relevant to the proposed cognitive pathways linking AI Literacy with self-reported higher-order capabilities. Practically, the findings highlight the potential relevance of integrating AI-related learning opportunities, thinking-oriented instruction, and supportive technological environments in vocational education. Because Complex Problem-Solving Skills were assessed through self-report, however, these findings should not be interpreted as evidence of objectively demonstrated improvement in students’ complex problem-solving performance.

## Data Availability

The participant-level datasets generated and/or analyzed during the current study are not publicly available because of participant privacy and ethical restrictions but may be made available from the corresponding author on reasonable request and subject to applicable ethical and institutional requirements.
